# Awareness, Familiarity, and Pharmacist Trust: A Structural Equation Model Analysis

**DOI:** 10.1177/87551225211052411

**Published:** 2022-03-15

**Authors:** Bobbi Morrison, Todd A. Boyle, Thomas Mahaffey

**Affiliations:** 1 St. Francis Xavier University, Antigonish, Nova Scotia, Canada

**Keywords:** practice, pharmacist/patient issues, management, trust, partial least squares, public awareness

## Abstract

**Background:** Trust in health care professionals is critical in the health care system and is needed for a patient to seek care, reveal sensitive information, and follow a specified treatment plan, among other things. **Objective:** To better understand trust in community pharmacists, this research develops a model of how patient awareness of the different community pharmacy roles (role awareness) and pharmacist familiarity influences pharmacist trust. **Methods:** A survey of pharmacy patients in Nova Scotia, Canada, occurred in November and December 2019, with quota sampling used to achieve representativeness by age, gender, and household income. A total of 640 usable surveys were obtained. Consistent partial least squares was deployed to test and refine the model. **Results:** Overall, the final model highlights that both role awareness and pharmacist familiarity influence patient assessments of pharmacist trust and explains 38.7% of its variance. Pharmacist familiarity has a stronger influence than role awareness on pharmacist trust. Results of the consistent partial least squares multigroup analysis found no statistically significant differences in the model based on patient gender. **Conclusion:** This research provides a means to capture interpersonal trust in community pharmacists and identifies 2 key determinants of such trust. This research also provides guidance on how to assess pharmacist trust, the value of patients knowing their pharmacist, and the value of patient awareness of the roles of the various professionals behind the counter. Such knowledge will help pharmacy managers, associations, and regulatory authorities develop evidence-informed plans to assess, rebuild, and sustain trust.

## Background

In health care contexts, trust has been argued to be central to medical relationships,^1^ as trust is required for a patient to seek care, reveal information to the health care provider, adhere to the recommended treatment plan,^1^ and helps legitimize the overall health care system.^2^ At its most basic level, trust may be thought of as “a willingness to rely upon an exchange partner in whom one has confidence”^3^ and is something that helps individuals make decisions in conditions of vulnerability or risk.^4^ Within the community pharmacy setting, research on trust has focused on various aspects of pharmacy practice and management. More common areas of research include trust in pharmacists and its impact on customer satisfaction, pharmacy selection and loyalty,^5^ antecedents of pharmacist trust,^6,7^ trust in the pharmacy—drug wholesale relationship,^8^ and pharmacist trust compared with and between other health care professionals,^9^ including well-known polls.^10^

In health care contexts, interpersonal trust is the type of trust that exists between patients and health care providers including doctors, nurses, and pharmacists. Hall et al^1^ propose 5 dimensions of interpersonal trust, specifically fidelity (ie, caring for the individual’s welfare and interests), competence (ie, making correct decisions and avoiding mistakes), honesty (ie, telling the truth and avoiding intentional falsehoods), confidentiality (ie, proper use of sensitive information), and global trust (ie, aspects of trust that are irreducible and not subject to dissection). While originally envisioned as composed of these distinct dimensions, subsequent research focused on physician trust identified interpersonal trust as unidimensional but composed of various items from these dimensions.^11^ Awareness and knowledge have been noted to be important prerequisites for interpersonal trust.^12^ Similarly, familiarity with the target of the trust has been conceptualized as an important factor in interpersonal trust.^13^

Within a pharmacy context, McMillan et al^14^ highlight that many studies have examined patient and public awareness of various aspects of pharmacy practice, such as the provision of pharmacy services and the role of the community pharmacist. Regarding the role of familiarity, investigations have linked patient familiarity with trust in pharmacists,^7,15^ but empirical research that explores how patient familiarity with their community pharmacist and awareness of community pharmacy roles impacts trust in their community pharmacist is still lacking. The need to better understand the relationship between role awareness, pharmacist familiarity, and pharmacist trust is especially important in recent years given, for example, the move away from dispensary models of care to expanded scope of pharmacy practice and its resulting changes to staff roles and responsibilities.

This research develops a model of how patient awareness of community pharmacy roles and familiarity with their community pharmacist influence pharmacist trust. Additionally, because gender differences have been observed in the extent to which health care providers are trusted^16,17^ and the extent to which receiving health services at a pharmacy are preferred,^18^ this research also explores model differences based on gender.

## Objectives

- Identify current levels of role awareness, pharmacist familiarity, and pharmacist trust among patients.
- Develop a model of how patient awareness of community pharmacy roles and familiarity with their community pharmacist influence pharmacist trust.
- Explore model differences based on patient gender.

## Methods

### Instrument Development

This specific research formed part of a project that explored various issues related to community pharmacist trust in Nova Scotia, Canada. Data for this study were collected using an online questionnaire. Measures of Role Awareness and Pharmacist Familiarity were developed by the researchers in consultation with pharmacy professionals. To capture Role Awareness, 4 items were used to gauge respondent awareness of the different community pharmacy practitioners and their ability to distinguish among practitioners behind the pharmacy counter. To capture Pharmacist Familiarity, 5 items were developed to measure the extent to which respondents were familiar with the community pharmacists in the pharmacy they frequent most often. The physician-focused operationalization of interpersonal trust proposed by Hall et al^1,11^ was adapted and condensed by researchers to the context of community pharmacists. Pharmacist trust was captured as a unidimensional construct composed of 9 items. Five-point Likert-type scales, ranging from 1 (ie, strongly disagree) to 5 (ie, strongly agree), were used to capture the items related to role awareness, pharmacist familiarity, and interpersonal trust, as presented in Table 1. The survey instrument was pretested with 35 respondents to assess content validity, comprehension, and completion time (15 minutes).

**Table 1.** Survey Measures.

	Abbreviation	Dimension of interpersonal trust
Pharmacist trust		
My pharmacist provides all the care I expect	FIDEXPT	Fidelity
My pharmacist only thinks about what is best for me	FIDME	Fidelity
My pharmacist’s skills are not as good as they should be*	COMPSKLRV	Competence
My pharmacist is extremely thorough and careful	COMPCARE	Competence
My pharmacist is totally honest	HONTOTAL	Honesty
I have no concerns about my pharmacist’s ability to keep my information private	CONFPRIV	Confidentiality
I worry that people can overhear me when I ask my pharmacist questions or provide information about my health*	COMFHRRV	Confidentiality
I completely trust my pharmacist about my medication decisions	GBLDECS	Global trust
All in all, I have complete trust in my pharmacist	GBLCPL	Global trust
Role awareness		
I am aware that it is possible for different types of pharmacy professionals to be working behind the pharmacy counter	ROLETYPE	
I can tell who the pharmacist is	ROLEPHARM	
I can tell who the pharmacy technician is	ROLETECH	
I am confident I know the difference between what a pharmacist and a pharmacy technician can do	ROLECONFID	
Pharmacist familiarity		
I interact with the same pharmacist(s) each time I visit the pharmacy	FMINTER	
I consider one pharmacist to be “my pharmacist”	FMRONE	
I know the pharmacist(s) at my pharmacy well	FMWELL	
I recognize the pharmacist(s) from my pharmacy when I see them in my community	FMRECOG	
I do not know the pharmacist(s) at my pharmacy by name*	FMNAMRV	

* Item reverse-coded for the model.

### Sample Selection

The questionnaire was prepared in English using Qualtrics software. Quota sampling was used to achieve representativeness by age, gender, and household income. Respondents aged 18 years and older living in the province of Nova Scotia were recruited using the third-party survey sampling company Dynata, following their recruitment protocols,^19^ and invited to participate in the online questionnaire. Participants were provided with a link to the Qualtrics survey directly by Dynata. Responses were anonymous; no personally identifiable information was collected by the researchers. Ethical approval for the study was provided by the St. Francis Xavier University Research Ethics Board.

### Proposed Model

**Figure 1.**
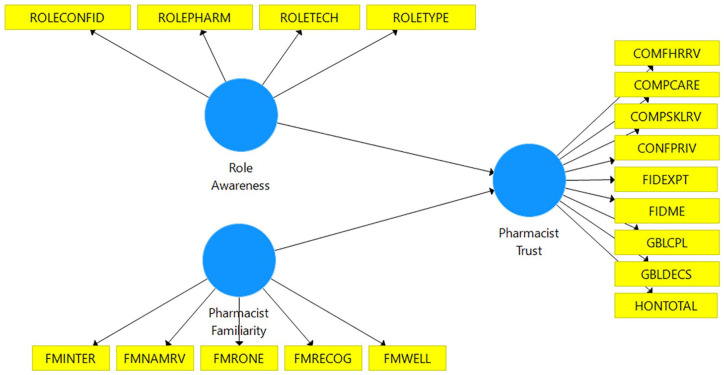
Proposed model of pharmacist trust. Refer to Table 1 for expansions for the abbreviations.

The model proposes that awareness of the various pharmacy roles (Role Awareness) and familiarity with the pharmacist (Pharmacist Familiarity) influences pharmacist trust. It is proposed that the patient’s awareness of the different pharmacy roles, such as the ability to distinguish between a pharmacist and technician, and the extent that the patient has interacted with their pharmacist will help increase understanding of expected pharmacist behaviors and, therefore, influence aspects of trust, especially those most apparent through patient-pharmacist interactions. A proposed model of the relationship between role awareness, pharmacist familiarity, and pharmacist trust is presented in Figure 1.

### Partial Least Squares (PLS) Analysis

IBM SPSS Statistics Version 26 was used to compute the descriptive statistics. PLS using SmartPLSv3^20^ was selected to test the proposed model. PLS is a variance-based structural equation modeling (SEM) estimation technique that is widely used in the social and behavioral sciences.^21^ As highlighted by Benitez et al,^21^ PLS “has become a full-fledge estimator of SEM that can deal with reflective and causal-formative measurement models, as well as composite models. Moreover, it can be applied to confirmatory, explanatory, exploratory, descriptive, and predictive research.” Given the absence of causal models that explore the relationship between pharmacist familiarity and interpersonal trust, and the lack of research that explores such constructs in a community pharmacy context, this research was deemed to be exploratory in nature and, as such, PLS was selected as the most appropriate estimating technique for model testing and refining.

Consistent with social science research and common for attitude measures, the 3 constructs of the PLS model were considered to be reflective in nature. For example, it is assumed that high levels of pharmacist trust will be reflected in its associated manifest variables. As outlined by Hair et al,^22^ manifest variables within each latent variable should be highly correlated with one another, manifest variables can be left out as long as sufficient reliability remains, and each latent variable should be composed of at least 3 manifest variables.

### Measurement Model Assessment

As such, the consistent PLS (PLSc) algorithm was applied given the presence of reflective constructs. The PLSc analysis was composed of 3 major steps: measurement model assessment, PLSc multigroup analysis, and structural model assessment. The first step of the PLSc analysis was the assessment of the quality of the measurement model (ie, relationship between the manifest variables and their associated latent variable). This included an examination of the average variance extracted, item loadings, internal consistency, and discriminant validity.

The commonly recommended average variance extracted (AVE) threshold value of 0.5 was used to assess the model’s convergent validity. Though the commonly accepted benchmark for manifest item loadings is 0.708, lower loadings may be accepted when newly developed scales are employed,^23^ as was the case in this study. Hair et al^24^ also advise that loadings between 0.40 and 0.70 should only be deleted when there is a corresponding increase in the AVE of the latent construct above the required threshold of 0.5. Therefore, items with outer loadings less than the suggested threshold of 0.708 were retained if the AVE was within an acceptable range. Internal consistency of the model’s latent constructs were evaluated using the composite reliability and rho_A with a threshold of 0.7. Discriminant validity was determined with the heterotrait-monotrait ratio (HTMT)^25^ using a threshold of HTMT <0.85.^21^

### PLSc Multigroup Analysis

The second step in the analysis was a PLSc multigroup analysis (MGA) to explore any differences in the model between the gender groups. This occurred through running the PLSc MGA functionality that spilt the data based on gender, compared the split datasets, and provided *P* values to determine any differences in the outer loadings and path coefficients between the datasets.

### Structural Model Assessment

The third step in the PLS analysis was an assessment of the structural model. Collinearity was first investigated using the variance inflation factor and compared with the maximum threshold of 5.^26^ PLSc bootstrapping was then performed to test for the significance and size of latent variable paths. Statistical significance of the latent variable paths was assessed using critical *t* values of 1.96 (*P* ≤ .05) and 2.58 (*P* ≤ .01). Coefficients of determination (*R*^2^) was then used to evaluate the in-sample predictive power of the model, with the common benchmark of 0.10 applied in order for the variance explained to first be deemed acceptable. This was followed up by an examination of the strength of the *R*^2^ using the benchmarks of 0.19 (ie, weak), 0.33 (ie, moderate), and 0.67 (ie, strong).^27^ The benchmarks of 0.02 (ie, small), 0.15 (ie, medium), and 0.35 (ie, large) were applied to assess the effect size (*f*^2^) of each independent variable’s contribution to the dependent variable’s *R*^2^.^28^

## Results

### Sample Characteristics

Survey administration and data collection occurred throughout November and December of 2019. A soft launch with 100 respondents was initially undertaken. Detecting no issues with soft launch responses, responses from those who completed the survey during the soft launch were retained in the final data set. The average survey completion time was 16 minutes. An initial sample size of 780 respondents remained after the data were cleaned to remove incomplete surveys and speeders (ie, completion time of <7 minutes). To ensure some recent interaction with a community pharmacist, respondents for this study were required to have had at least one prescription filled at a community pharmacy within the past 6 months. This requirement reduced the usable sample size to 640 respondents, yielding a margin of error of 3.81% at 95% confidence.^29^ Sample characteristics are specified in Table 2.

**Table 2.** Respondent Demographics.

	N	%
Gender		
Female	330	51.6
Male	306	47.8
Nonbinary	1	0.2
Not listed	2	0.3
Prefer not to answer	1	0.2
Total gender	640	100.0
Age (years)		
18-24	57	8.9
25-34	73	11.4
35-44	86	13.4
45-54	107	16.7
55-64	135	21.1
65+	182	28.4
Total age	640	100.0
Education		
Less than high school	16	2.5
High school	118	18.4
Some college/university	128	20.0
College/university degree/diploma	299	46.7
Postgraduate degree	73	11.4
Prefer not to answer	6	0.9
Total education	640	100.0
Household income		
Under $10 000	13	2.0
$10 000-$29 999	97	15.2
$30 000-$59 999	173	27.0
$60 000-$99 999	161	25.2
$100 000-$149 999	119	18.6
$150 000+	52	8.1
Prefer not to answer	25	3.9
Total household income	640	100.0
Household size		
1	130	20.3
2	285	44.5
3	110	17.2
4	78	12.2
5	33	5.2
Prefer not to answer	4	.6
Total household size	640	100.0
Prescriptions filled in the last 6 months		
1 to 3	313	48.9
4 to 6	160	25.0
7 to 9	67	10.5
10 or more	100	15.6
Total prescription filled	640	100.0
Pharmacy location		
Urban	198	30.9
Suburban	190	29.7
Rural	249	38.9
Prefer not to answer	3	.5
Total location	640	100.0

### Levels of Awareness, Familiarity, and Trust

Respondents were asked about their ability to distinguish among practitioners behind the pharmacy counter and the extent to which they were familiar with the community pharmacists in the pharmacy they frequent most often. Examining the individual items (ie, survey questions), most respondents were aware that different types of pharmacy professionals worked behind the pharmacy counter and had a moderate degree of familiarity with the pharmacists at the community pharmacy they visited most frequently. Overall, respondents placed a fairly high degree of trust in their pharmacists. Levels of awareness, familiarity, and trust among the survey respondents are presented in Table 3.

**Table 3.** Patient Familiarity and Trust.

	N	%	Mean^a^	Standard deviation
Role awareness				
I am aware that it is possible for different types of pharmacy professionals to be working behind the pharmacy counter	634	99.06	4.24	.81
I can tell who the pharmacist is	634	99.06	3.66	1.10
I can tell who the pharmacy technician is	627	97.97	3.32	1.11
I am confident I know the difference between what a pharmacist and a pharmacy technician can do	624	97.50	3.51	1.13
Pharmacist familiarity				
I interact with the same pharmacist(s) each time I visit the pharmacy	637	99.53	3.30	1.16
I consider one pharmacist to be “my pharmacist”	639	99.84	3.05	1.21
I know the pharmacist(s) at my pharmacy well	639	99.84	3.19	1.21
I recognize the pharmacist(s) from my pharmacy when I see them in my community	635	99.22	3.51	1.18
I do not know the pharmacist(s) at my pharmacy by name	638	99.69	3.08	1.30
Pharmacist trust				
My pharmacist provides all the care I expect	637	99.53	4.13	.75
My pharmacist only thinks about what is best for me	619	96.72	3.80	.84
My pharmacist’s skills are not as good as they should be	607	94.84	2.19	1.04
My pharmacist is extremely thorough and careful	620	96.88	4.12	.77
My pharmacist is totally honest	589	92.03	4.00	.81
I have no concerns about my pharmacist’s ability to keep my information private	634	99.06	4.08	.99
I worry that people can overhear me when I ask my pharmacist questions or provide information about my health	636	99.38	2.93	1.22
I completely trust my pharmacist about my medication decisions	631	98.60	4.04	.81
All in all, I have complete trust in my pharmacist	630	98.44	4.07	.82

a Scale ranged from 1 (strongly disagree) to 5 (strongly agree).

### Measurement Model Results

Assessment of the PLS measurement model involved analyses of internal consistency/composite reliability, convergent validity, and discriminant validity. The manifest item loading threshold of 0.708 (or between 0.4 and 0.708 depending on AVE) and AVE threshold of 0.5 for each latent construct was used to assess indicator reliability. Based on this analysis, 3 items were eventually dropped as their loadings were <0.4 (ie, COMFHRRV = .30, ROLETYPE = .35, COMPSKLRV = .39). CONFPRIV was dropped given its low loading (ie, .41) and resulting improvement in AVE (ie, increase from .54 to .58). With these manifest variables removed, the 3 latent variables were all above the recommended AVE threshold of 0.5 and composite reliability threshold of 0.7. Results of the analysis also indicated sufficient discriminant validity with the HTMT values for all 3 latent variables well below the 0.85 cutoff. Table 4 presents the reliability and discriminant validity of the revised measurement model.

**Table 4.** Discriminant Validity and Reliability.

Construct	Reliability			Discriminant validity (HTMT)	
	AVE	CR	Rho_A	Role awareness	Pharmacist familiarity
Role awareness	.55	.79	.80		
Pharmacist familiarity	.60	.88	.89	.46	
Pharmacist trust	.58	.89	.89	.42	.60

Abbreviations: AVE, average variance extracted; CR, composite reliability; HTMT, heterotrait-monotrait ratio of correlations.

### PLS Multigroup Analysis Results

Prior to testing the structural model, a PLSc MGA occurred to explore any differences in the model between the 2 gender groups. Results of the PLSc MGA indicated all outer loadings and path coefficients possessed *P* values >.05. No statistically significant differences between the 2 gender groups were identified. As a result, there are no differences in the model based on patient gender.

### Structural Model Results

**Figure 2.**
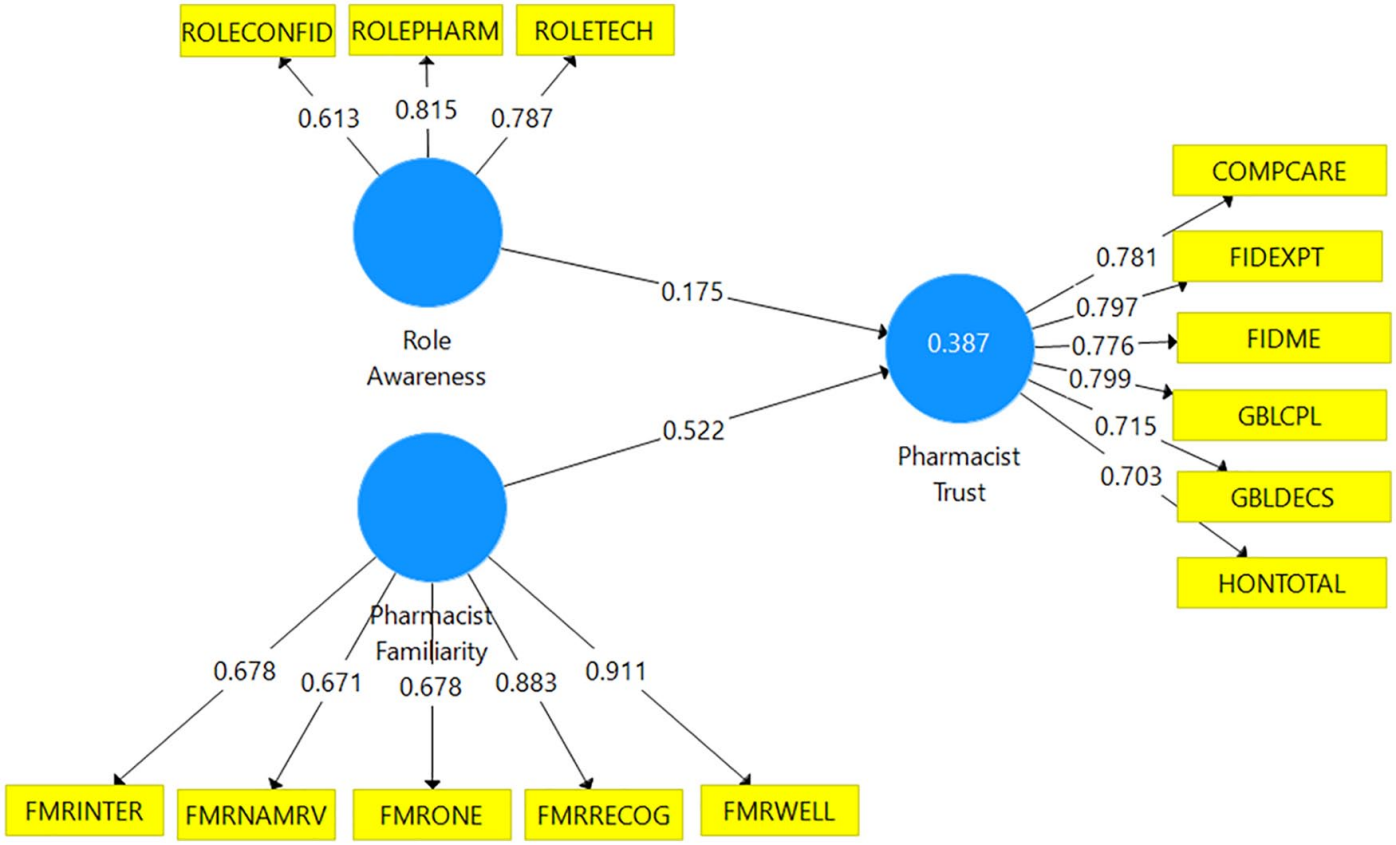
Final model of pharmacist trust. Refer to Table 1 for expansions for the abbreviations.

The PLSc bootstrapping results indicated that both paths were statistically significant (ie, *P* ≤ .01) and explained 38.7% of the variance in pharmacist trust. Effect sizes of model paths included low (*f*^2^ = 0.04) for Role Awareness → Pharmacist Trust and large (*f*^2^ = 0.35) for Pharmacist Familiarity → Pharmacist Trust. There were no issues of collinearity among model constructs observed as variance inflation factor statistics ranged from 1.3 to 3.3 and, therefore, well below the threshold of 5. The final model of pharmacist trust that includes the variance explained, outer loadings, and path coefficients is presented in Figure 2.

## Discussion

Overall, the findings reinforce public opinion polling that indicates patients place a high degree of trust in pharmacists.^10^ Patients agreed that their pharmacist provides all the care that they expect, is extremely thorough and careful, is totally honest, and keeps their health information confidential. In addition, while the public appeared to understand that different types of pharmacy professionals may be working behind the pharmacy counter, identifying such individuals presented challenges to some patients. Consistent with findings by Kelly et al,^30^ the ability of patients to distinguish between the pharmacist and pharmacy technicians and their confidence in their role differences scored lower when compared with patients’ general understanding that multiple professionals may be present behind the counter. Patients had moderate familiarity with their community pharmacists. Respondent familiarity with pharmacists was observed to be highest in their ability to recognize their pharmacist within their community (ie, outside of the pharmacy) and interaction with the same pharmacist(s) each time they visited the pharmacy. Patients tended to agree that they know their pharmacist well, yet not necessarily by name. No equivalent measures for pharmacist familiarity could be found for comparison.

Overall, the final model highlights that role awareness and pharmacist familiarity explains 38.7% of the variance in pharmacist trust. These findings reinforce^7^ and extend^6,7^ our understanding of the antecedents of pharmacist trust. Specifically, the final model revealed that pharmacist familiarity has a stronger influence than role awareness in assessments of pharmacist trust. Separate from the influence of institutional trust on a patient’s relationship with their pharmacy, interpersonal familiarity provides opportunities for patients to directly experience key elements of their pharmacist such as honesty, care, and thoroughness. Attributes of a provider-client relationship, including frequency of interaction and relationship duration, influence psychological outcomes such as trust.^31^ Thus, results also highlight the importance of maximizing patient-pharmacist interaction so that patients can come to recognize the pharmacists with whom they work and to potentially form relationships with “their” pharmacist. Such interaction is consistent with the profession’s desired transition to more cognitive services and is facilitated by recent practice environment initiatives that delegate purely dispensary-related tasks to newly regulated pharmacy technicians. Overall, results show that recent changes to the practice environment provide increased opportunities for greater familiarity between pharmacists and patients and this familiarity, in turn, has the potential to pay dividends in increased interpersonal trust.

The resulting model has important implications to both pharmacy research and practice. While interpersonal trust has been explored in health care generally, empirical research focused on the various aspects of interpersonal trust within a community pharmacy context is limited. Though interpersonal trust has been measured in multiple studies, and antecedents have been modelled,^6,7^ no study operationalized trust in the same way, making comparability of findings difficult and highlighting the need for consistent measurement. Building on Hall et al’s^1,11^ interpersonal trust in health care context, this research presents a unidimensional and condensed measure of interpersonal trust between patient and pharmacist that is parsimonious, reliable, and valid. This study’s results necessitated dropping items related to confidentiality, indicating that confidentiality may be better captured as a separate construct from interpersonal trust within a community pharmacy context. Additionally, despite support in the literature for differences in pharmacist trust based on gender, no such differences were found for the patients in this particular study.

This research also has important implications to pharmacy practice. In addition to reinforcing the value of moving to greater pharmacist-patient interaction via expanded scope of practice and regulation of pharmacy technicians, as mechanisms for increasing interpersonal trust and thus improved patient retention, results also show the importance of increased employee (pharmacist) retention without which familiarity and awareness would be more difficult to achieve.

There are various situations where an assessment of the public’s interpersonal trust in community pharmacists as initiated by pharmacy managers, pharmacy associations, or pharmacy regulatory authorities may be beneficial. For example, given the prevalence of medication incidents within health care, community pharmacy managers may find themselves needing to assess, rebuild, or sustain public trust in their pharmacists as a result of a severe and widely publicized medication incident that may have occurred within their pharmacy or jurisdiction. Likewise, a pharmacy association or pharmacy regulatory authority may want to assess public trust in pharmacists across their jurisdiction to help inform decisions about standards of practice, communication strategies, and deployment plans related to the introduction of new expanded pharmacy services. This research provides guidance to these various stakeholders as to how interpersonal trust in pharmacists should be assessed, the value of patients knowing their pharmacist, and the value of patient awareness of the roles of the various professionals behind the counter.

There are a number of limitations to this research that may also represent valuable future research opportunities. The PLS model captured pharmacist trust as a single latent variable with reflective manifest variables. However, given that research has proposed trust as comprising elements of fidelity, competence, confidentiality, honesty, and global trust, future research should explore each of these elements as separate latent variables. For example, using PLS, future research should assess whether trust is best captured as a single latent variable (as in this research), as 5 separate latent variables, or as a second-order construct. Along with such an analysis, further consideration is needed if such latent variables are better captured as formative versus reflective.

This model only explored the female and male gender groups. Given their very small sample size, other gender groups identified in the sample could not be analyzed. As a result, future research should explore whether views of trust in community pharmacists differs among other gender groups not represented in this study. Future research is also needed to explore how other variables may affect the model, such as the type of interaction that the patient and pharmacist had (eg, simple greeting/exchange vs clinical encounter vs patient education), and number of conditions/medications, and control/severity of a disease.

## Conclusion

Trust in health care professionals, such as community pharmacists, is needed for a patient to first seek care, reveal sensitive health-related information, and adhere to a recommended treatment plan.^1^ The resulting PLS model provides guidance on how to assess pharmacist trust, the value of patients knowing their pharmacist, and the value of patient awareness of the roles of the various professionals behind the counter. Such knowledge will help pharmacy managers, associations, and regulatory authorities better develop plans to assess, rebuild, and sustain trust when needed.
